# Massive deregulation of miRNAs from nuclear reprogramming errors during trophoblast differentiation for placentogenesis in cloned pregnancy

**DOI:** 10.1186/1471-2164-15-43

**Published:** 2014-01-18

**Authors:** Md Munir Hossain, Dawit Tesfaye, Dessie Salilew-Wondim, Eva Held, Maren J Pröll, Franca Rings, Gregor Kirfel, Christian Looft, Ernst Tholen, Jasim Uddin, Karl Schellander, Michael Hoelker

**Affiliations:** 1 Institute of Animal Science, Animal Breeding and Husbandry Group, University of Bonn, Endenicher Allee 15, 53115 Bonn, Germany; 2 Animal Breeding & Genetics, Bangladesh Agricultural University, Mymensingh 2202 Bangladesh; 3 Institute for Cell Biology, University of Bonn, Ulrich-Haberland-Strasse 61a, 53121 Bonn, Germany

**Keywords:** miRNAs, IVP, SCNT, Reprogramming, Placenta, Trophoblast, Differentiation, Bovine

## Abstract

**Background:**

Low efficiency of Somatic Cell Nuclear Transfer (NT) has been widely addressed with high incidence of placental abnormalities due to genetic and epigenetic modifications. MiRNAs are shown to be major regulators of such modifications. The present study has been carried out to identify the expression patterns of 377 miRNAs, their functional associations and mechanism of regulation in bovine placentas derived from artificial insemination (AI), in vitro production (IVP) and NT pregnancies.

**Results:**

This study reveals a massive deregulation of miRNAs as chromosomal cluster or miRNA families without sex-linkage in NT and in-vitro derived IVP placentas. Cell specific localization miRNAs in blastocysts and expression profiling of embryos and placentas at different developmental stages identified that the major deregulation of miRNAs exhibited in placentas at day 50 of pregnancies is found to be less dependent on global DNA methylation, rather than on aberrant miRNA biogenesis molecules. Among them, aberrant AGO2 expression due to hypermethylation of its promoter was evident. Along with other factors, aberrant AGO2 expression was observed to be associated with multiple defects in trophoblast differentiation through deregulation of miRNAs mediated mechanisms.

**Conclusion:**

These aberrant miRNA activities might be associated with genetic and epigenetic modifications in abnormal placentogenesis due to maldifferentiation of early trophoblast cell lineage in NT and IVP pregnancies. This study provides the first insight into genome wide miRNA expression, their role in regulation of trophoblast differentiation as well as abnormal placental development in Somatic Cell Nuclear Transfer pregnancies to pave the way to improve the efficiency of cloning by nuclear transfer.

## Background

Animal cloning is a break-through technology with emerging potential applications in agricultural and biomedical research. However, the technology is hindered by very low rates of healthy live births attributed to high incidences of placental abnormalities leading to embryonic losses [1,2]. The major sources of these abnormalities is thought to be genetic and epigenetic modifications occurred due to improper reprogramming of donor cells after nuclear transfer leading to post-implantation lethality or abnormality [3-5]. Key mechanisms underlying this aberration are DNA methylation, histone remodeling and telomere maintenance, which are involved in the control of gene expression, X chromosome inactivation and genomic imprinting [6,7]. However, all these mechanisms are not contributing equally to the embryonic and extra-embryonic lineage [8,9]. Specially, genomic imprinting was found to be less or not dependent on DNA methylation for its somatic maintenance in placentas than in the embryos. Whereas, placenta-specific imprinting has been shown to be more dependent on repressive histone modifications as well as regulation by non-coding RNAs [7,10]. Among the non-coding RNAs, a recently discovered class of small RNAs, namely miRNAs appeared as important regulators of gene expression at the transcriptional or post-transcriptional level. They are found to play important roles including but not restricted to cell proliferation, apoptosis, diseases and differentiation during mammalian development [11,12]. Moreover, they are targeted by epigenetic modifications in which some imprinted miRNAs were found to undergo subsequent epigenetic reprogramming in mouse embryos [13,14]. However, the patterns of miRNAs expression contributing to the widely addressed placental abnormalities along with genetic and epigenetic modifications in the IVP or NT placentas are yet to be discovered. Therefore, the main objective of this study was to investigate the expression of regulatory non-coding miRNAs in bovine day-50 placentas derived from pregnancies established after AI or transfer of IVP and SCNT derived bovine embryos. For this, the expression of 377 miRNAs was investigated using a quantitative real time PCR array technology in placentas of these three origins as well as donor fibroblast cells used for SCNT. Furthermore, selected differentially expressed miRNAs were analyzed for their expression in expanded blastocysts, day-16 elongated embryos as well as placentas at day 50 and day 225 of pregnancies from SCNT, IVP and AI embryos. Finally, methylation status and activities of miRNAs processing molecules were also studied in those samples.

## Results

### Differential miRNA expression in day-50 placentas of different sources of pregnancy

**Figure 1:**
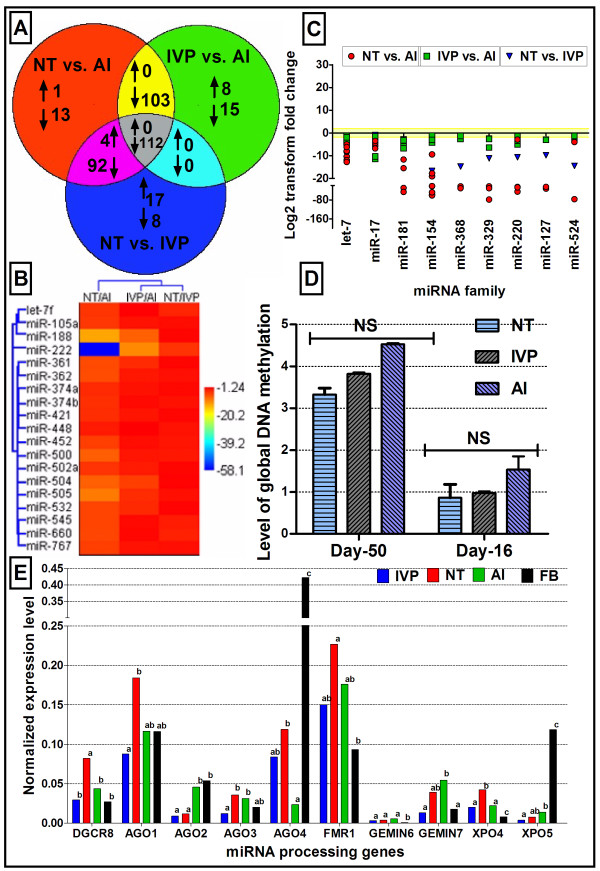
**Number and types of differentially expressed miRNAs in different sources of placenta together with their relation to global methylation status and expression of miRNA processing genes. (A)** Venn diagram of the distribution and the number of differential miRNA expression between and specific to different types of day-50 placentas. Each circle represents the number of differentially expressed miRNAs between two placenta types out of 377 analyzed miRNAs. For example, NT vs. AI represents the number of differentially regulated miRNAs (↓ denotes down regulation and ↑ denotes upregulation) in the NT placentas compared to that of AI. **(B)** Hierarchical cluster (log2 fold change) of NT, IVP and AI day-50 placentas compared to each other and characterization of differentially expressed X-linked miRNAs. **(C)** Family wise differential expression of miRNAs in different sources of day-50 placentas. Log2 fold change of 43 miRNAs belong to 9 miRNA families which were deregulated in different sources of placenta were plotted to visualize the family wise expression differences in the placentas of different sources of pregnancies. **(D)** Global DNA methylation (%) in day-16 elongated embryos and day-50 placenta of different sources of pregnancy. **(E)** Significant differential expression of 10 miRNA processing genes in different sources of placentas and donor fibroblast cells.

The expressions of 377 miRNAs were compared between placentas at day 50 of pregnancy derived from NT (n = 3), IVP (n = 3) and AI (n = 3) embryos (Figure 1A). Among the 377 miRNAs, 278 miRNAs were found to be downregulated in NT placentas with 2 or more fold change with P-value ≤ 0.05, while only 5 miRNAs (miR-527, miR-608, miR-637, miR-649 and miR-938) were found to be upregulated in the same placenta group compared to the placentas of AI pregnancies. Out of all downregulated miRNAs, 116 were unique to the NT placentas compared either to AI or IVP placentas. On the other hand, a total of 191 miRNAs were found to be differentially expressed in the NT placentas compared to their IVP counterparts. Among these, only 21 miRNAs were upregulated and the remaining 170 miRNAs were found to be downregulated. Comparison of IVP and AI derived placentas revealed differential expression of 238 miRNAs, of which 8 miRNAs (miR-122, miR-302a, miR-302b, miR-302c, miR-525-3p, miR-526b, miR-590-3p and miR-944) and 230 miRNA were found to be up and down regulated, respectively. Of these downregulated 230 miRNAs, 215 were also found to be down regulated in NT placentas compared to IVP derived ones.

### Comparative expression of miRNA between donor cells and NT day-50 placentas

To investigate the source-of-origin specific miRNA expression pattern, the profiles of same 377 miRNAs have been compared between NT placentas and the fibroblast donor cells. A total of 209 miRNAs were found to be differentially expressed between these two groups by fold change 2 or more with *P value* ≤0.05. Among these, 185 and 24 miRNAs were found to be up and down regulated in NT placentas compared to fibroblast donor cells, respectively.

### Chromosomal allocation of deregulated miRNAs in NT and IVP placentas

**Figure 2:**
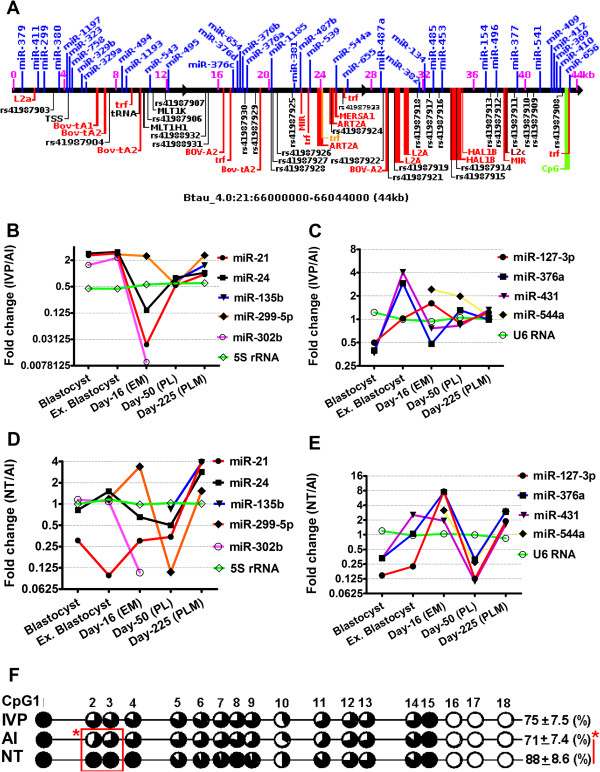
**Localization of miRNA in the chromosome as polycistronic cluster and expression of trophectoderm/placenta specific/imprinted miRNAs in different stages of embryos and placentas from different sources of pregnancies. A**: Genomic region (44 kb) of bovine chromosome 21 harboring at least 3 big clusters of miRNAs which are down regulated in day-50 NT and IVP placentas compared to that of AI. Text in blue above the black line (forward genomic strand) represents the residing miRNAs; number in pink represents range and scale of the region in kilo bases (kb), number in black starting with rs- denotes dbSNPs, others are different types of transposone (red or orange) namely type I line/SINE, type II, tandem repeats (trf) and pseudo transfer RNA (tRNA). **B**-**E**: Expression pattern (in fold change) of the candidate trophectoderm/placenta specific **(B)** and imprinted miRNAs **(C)** in blastocyst, expanded blastocyst, day-16 elongated embryo, day-50 placenta and day-225 placentome derived from IVP pregnancies compared to that from AI. Expression pattern (in fold change) of the candidate trophectoderm/placenta specific **(D)** and imprinted miRNAs **(E)** in blastocyst, expanded blastocyst, day-16 elongated embryo, day-50 placenta and day-225 placentome derived from NT pregnancies compared to that of AI pregnancies. **F**: Methylation status of *AGO2* promoter region in the day-50 placentas from different sources of pregnancies. Note the degree of methylation in NT placentas significantly differed from AI placentas.

The chromosomal location of deregulated miRNAs and features of genomic regions were retrieved from miRBase v 14 and ENSEMBL genome browser (Btau_4.0 assembly, Ensembl release 63) [15]. Deregulated miRNAs in NT and IVP placentas were found to exhibit similar patterns and localizations on the chromosomes as polycistronic clusters. One of such genomic region has been identified in bovine chromosome 21 (btau21:66000000–66044000, 44 kb) harboring at least 3 clusters of miRNAs comprising more than 38 miRNAs (Figure 2A). Most of these clustered miRNAs were found to be downregulated in NT and some in IVP placentas compared to that of AI. Genomic sequence of these cluster were found to be devoid of any protein coding gene but enriched in several genomic variable elements including different types of transposone elements, (type I LINE, type I SINE, type II), tandem repeats (TRF), long terminal repeats (LTRs) and numerous SNPs (Figure 2A). However, most of these elements and SNPs were found to be residing out side the mature miRNA or precursor sequences. Moreover, family wise expression pattern of miRNAs was evident for differentially expressed miRNAs (Figure 1C). The top 43 miRNAs, which are downregulated in NT and IVP day-50 placentas, belong to 9 distinct miRNA families. Similarly, miRNAs sharing similar sequence (denoted and distinguish by a, b, c, etc.) were also found to be deregulated in the placentas in a similar manner. Interestingly, most of the miRNAs located on the bovine X-chromosome, were found not to be differentially regulated between contrasting types of placentas under investigation (Figure 1B). However, among the x-linked miRNAs, miR-188, miR-222, miR-504 and miR-505 were found to be downregulated in NT and IVP placentas compared to their AI counterparts.

### Localization of selected miRNAs in expanded blastocyst of NT, IVP and AI origin

**Figure 3:**
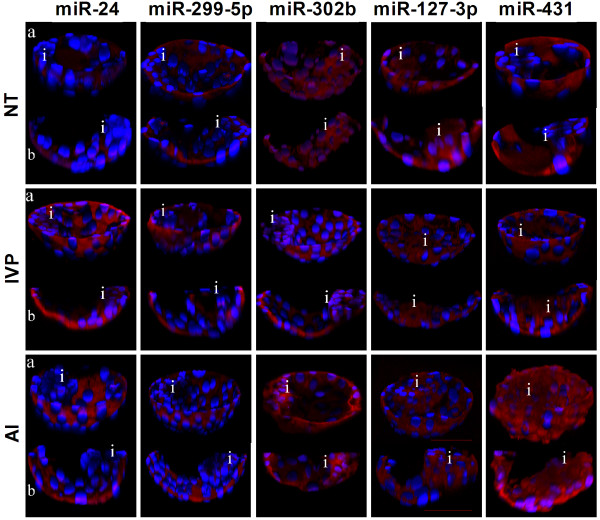
**Whole-mount in situ hybridization of miRNAs in in vivo, in vitro and NT expanded blastocysts.** MiRNAs are stained red and blue represents nuclear stain by DAPI. About half of the blastocyst comprising approximately middle region of the inner cell mass and corresponding trophectoderm has been visualized in the upper image **(a)** in each group as obtained by scanning the blastocyst at every 2 μm interval using laser scanning confocal microscope and projected in three dimensions (3D). Similarly lower image **(b)** for every group of embryos represents a 3D transverse region of upper and same blastocyst to visualize inner cell mass and trophectoderm clearly. Scale bar represents 50 μm and ‘i’ indicates the region of inner cell mass.

To identify spatial difference in miRNAs expression at earliest stage of development and existence of aberrant expression of miRNA between trophectoderms derived from different sources of blastocysts, candidate miRNAs were localized through whole mount in-situ hybridization. Among these candidate miRNAs, miR-24, and miR-299 were found to be intensively localized to the trophectoderm, while miR-302b to inner cell mass considering in-vivo (AI) derived expanded blastocyst (Figure 3). Expression of trophectoderm specific miRNAs was lower in NT blastocysts compared to AI counterparts. Two other miRNAs (miR-127 and miR-431), which are residing in the large imprinted chromosomal region were found to be similarly expressed in both the inner cell mass and the trophectoderm of AI blastocysts, while being aberrantly expressed in NT blastocysts. In NT blastocysts, miR-127 is almost depleted from the trophectoderm with no change in the inner cell mass. Results of in situ hybridization of miRNAs revealed aberrant miRNAs expression in the trophectoderm and inner cell mass of NT expanded blastocyst compared to their AI counterparts. Negative control probes have been used for all groups during hybridization and they were found to be negative (data not shown).

### Temporal expression pattern of candidate miRNAs in different sources of placenta

Expression profiling of candidate miRNAs considering in vivo, in vitro or NT originated day-7 blastocyst, expanded blastocyst, day-16 elongated embryos, day-50 placentas and day-225 placentomes revealed that the major deregulations were likely to happen in the NT placentas at day-50 of the pregnancy when compared to the AI placentas (Figure 2B-E). However, the extent of this deregulation in IVP placentas at day 50 was lower and showed very less difference compared to the placentas of AI at day 50 of pregnancy. Moreover, this deregulation of miRNAs found to be started as early as first cell lineage differentiation in nuclear transfer derived embryos and progressing to the later stage. This is more profound in the day-50 placentas, at which time point, most aberrant miRNAs expression is evident concomitant to redifferentiation for placentogenesis.

### DNA methylation status in different sources of elongated embryos and placentas

Global DNA methylation pattern has been investigated in elongated day-16 embryos and day-50 placentas derived from NT, IVP and AI pregnancies. Results revealed a global hypomethylation of DNA in all three groups (NT, IVP and AI) across these two stages of development (Day-16 elongated embryos and day-50 placentas) (Figure 1D). In contrast, there was no significant difference in global DNA methylation pattern comparing elongated day-16 embryos as well as day-50 placentas between NT, IVP and AI pregnancies.

### Expression pattern of miRNAs processing genes in IVP and NT placentas

**Figure 4:**
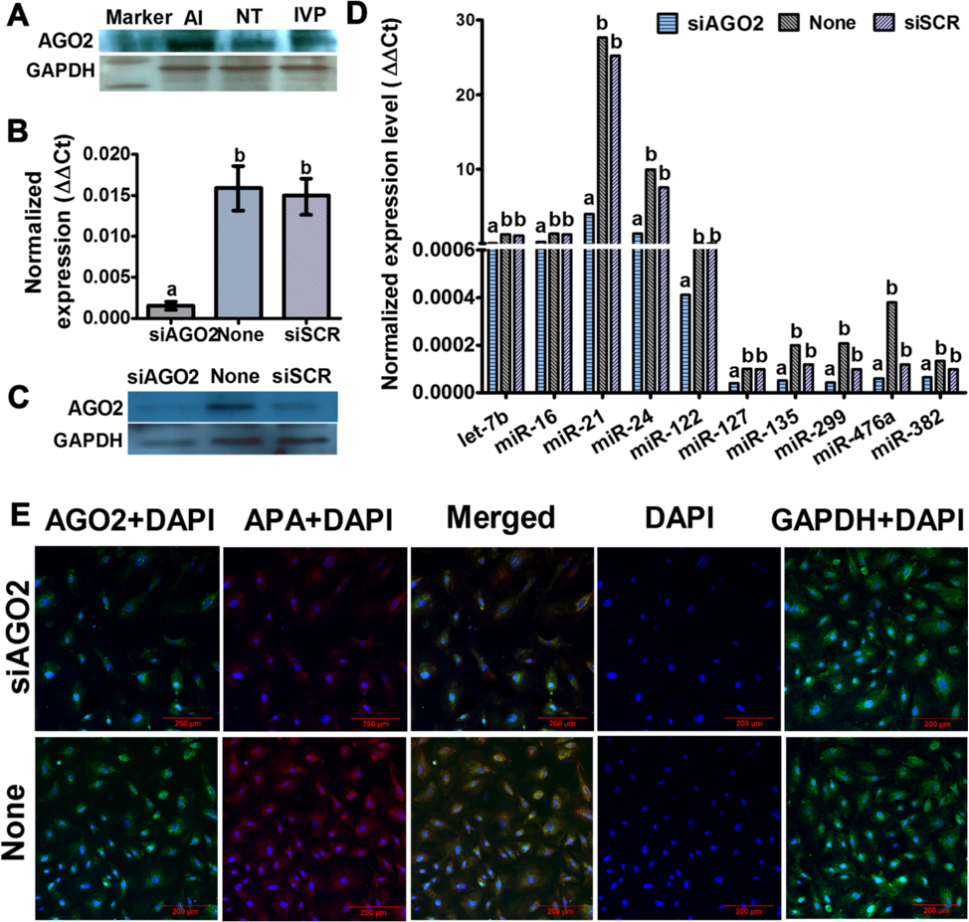
**Expression of *AGO2 *mRNA, protein and effect of knocking down of *AGO2 *mRNA in the regulation of miRNAs expression and trophoblastic stem cell differentiation. A**: Expression of AGO2 protein in the day-50 placentas from AI, NT and IVP derived pregnancies **(A)**. Expression of *AGO2* mRNAs **(B)** and protein **(C)** 48 hours after transfection of AGO2 siRNA (siAGO2), nontransfected control (None) and transfection of nonspecific scrambled siRNAs (siSCR) in the TBSs during their differentiation in vitro. **D**: Expression of selected candidate miRNAs in three groups of treated TBSs (siAGO2, none and siSCR). Images on the expression of AGO2 and the level of alkaline phosphatase activity (APA) in the same three groups of cells **(E)**.

Expression profiling of miRNA processing machinery genes in day-50 placentas from NT and IVP pregnancies compared to that of AI was performed (Figure 1E). Results revealed that all genes under study except *AGO2* were transcriptionally reprogrammed in NT compared to AI placentas. However, miRNA processing genes including *AGO2*, *AGO3*, *GEMIN7*, and *XPO4* were found to be deregulated in IVP placentas compared to their AI counterparts. *AGO2* (*EIF2C2*: eukaryotic transcription initiation factor 2C2) mRNA and protein (Figure 4A) were found to be down regulated in both NT and IVP day-50 placentas compared to that of AI.

### Status of AGO2 promoter methylation in different placentas

The DNA methylation status of 18 CpG sites in the CpG island of AGO2 promoter region were analyzed and results evidenced a significantly higher methylation status of the AGO2 promoter in NT placentas (89 ± 9.7%) compared to AI placentas (72 ± 2.7%) (Figure 2F). In IVP placentas, the promoter was methylated by 76 ± 7.52%. Moreover, two CpG sites located at 757 bp and 789 bp downstream of the transcription start site, respectively (Btau: 4.0-21:2371776) showed significantly increased DNA methylation (100% for both sites) in NT placentas compared to AI placentas (57% and 71%, respectively).

### Functional analysis of *AGO2* in differentiation of Trophoblastic stem cells (TBSs)

**Figure 5:**
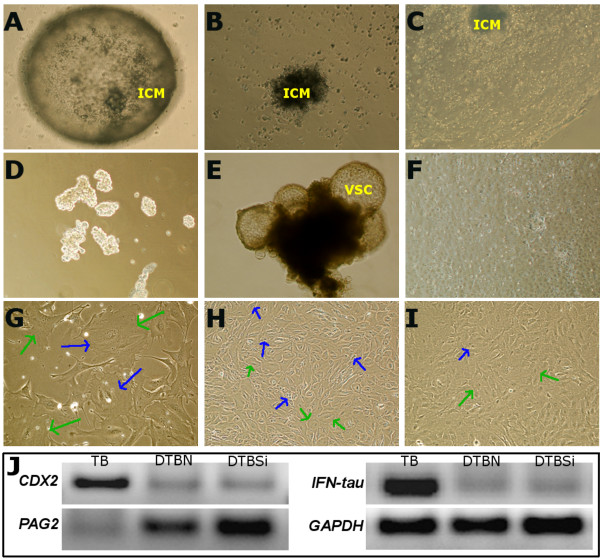
**Derivation, culture, validation and knocking down of *AGO2 *during differentiation of trophoblastic stem cells (TBSs).** Culture of blastocyst at day 12 **(A)**, derivation of TBSs at day 14 of culture **(B)**, Fully grown TBSs **(C and F)**, TBSs plated for subculture **(D)**, developing trophoblastic vesicles (VSC) **(E)**, differentiated TBSs into distinctive placental cells **(G)**, nontransfected differentiated TBSs **(H)** and differentiated TBSs transfected with AGO2 siRNA **(I)**. **J**: Expression of *Homeobox protein CDX-2, Interferon tau-(ifn-tau)*, *pregnancy*-*associated glycoproteins 2-PAG2* and *Glyceraldehyde 3-phosphate dehydrogenase-GAPDH* mRNAs (as control) in TBSs (TB), nontransfected differentiated TBSs (DTBN) and differentiated TBSs transfected with AGO2 siRNA (DTBSi). Blue arrow indicates binucleate cells and green arrow shows trophoblastic giant cells.

**Figure 6:**
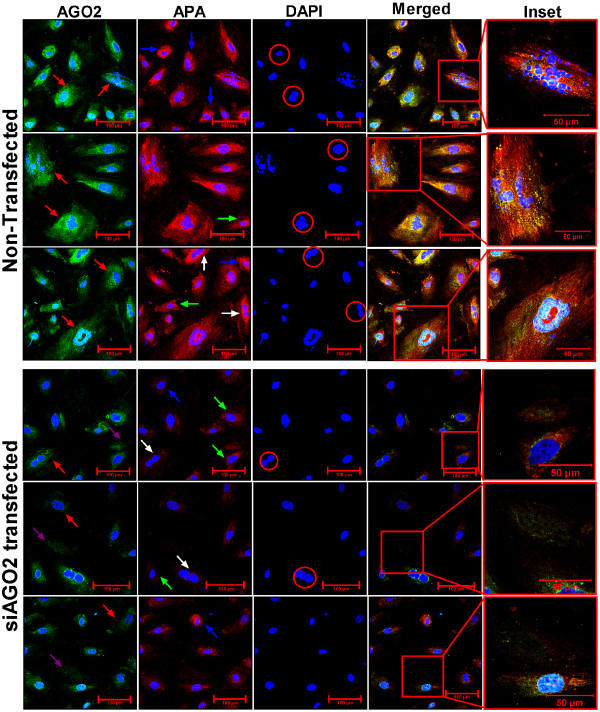
**Effect of knocking down of *AGO2 *mRNA in the regulation of trophoblastic stem cell differentiation.** Expression of AGO2 protein (green) 48 hours after transfection of AGO2 siRNA (siAGO2) and nontransfected control (None) in the TBSs during their differentiation in vitro. Trophoblastic giant cells, Glycogen cells and Spongiotrophoblast cells are indicated by red, blue and green arrowhead. White arrow and red circle indicates binucleate cells and endopolyploidy level identified by the blue nuclear stain (DAPI) in the images including inset. Scale bar: 100 μm (50 μm in the right column).

To substantiate, whether global deregulation of miRNAs is associated with defective differentiation of trophoblast cells leading to abnormal placentogenesis in the in-vitro model, TBSs were derived and used to induce knockdown of *AGO2* mRNA using sequence specific siRNA. The TBSs were derived from in vitro produced bovine blastocysts which were found to form a monolayer sheet of polygonal cells on type I collagen coated flasks (Figure 5C). Spherical multicellular vesicles of 100 to 900 μm in diameter were found to be continuously formed through accumulation of fluid under the cell sheet (Figure 5E). These vesicles, which resemble the trophectoderm of blastocysts, were used for subculture and subsequent knock-down experiments. The epithelial phenotype of TBSs was validated by the expression of *IFN-tau*, which is a marker of trophectoderm cells in ruminants (Figure 5J) (Martal et al. 1998; Roberts et al. 1999). The procedure of TBSs culture for in vitro differentiation in this experiment was modified from Nakano et al., (2002) and results revealed not only the differentiation of TBSs into binucleate cells (17% of total cell population) but also into distinct types of different mononucleated cells namely trophoblast giant cells (15%), spongiotrophoblast cells (8%), glycogen cells (10%), and syncytial trophoblast cells (8%) (Figures 6 and 4E). Differentiated TBSs were characterized by lower to almost no expression of *CDX2* and higher expression of *PAG2* by RT-PCR (Figure 5J).

### Effect of AGO2 knock down on in vitro differentiation of TBSs

Induced down regulation of miRNAs during in vitro TBS cell differentiation was achieved through knock down of AGO2 by siRNA technology. Specificity of siRNA mediated *AGO2* knock-down was confirmed by significant reduction of the corresponding mRNA by approximately 60% (Figure 4B) and resulted in reduced protein expression for the same gene as demonstrated by western blotting (Figure 4C) as well as immunocytochemistry (Figure 6) compared to the controls. Quantitative real time PCR was used to analyze changes with respect to expression of some candidate trophectoderm/placenta specific and imprinted miRNAs, which were found to be downregulated after *AGO2* knockdown (Figure 4D). Expression of TBSs cell differentiation and placental cell markers *(CDX2, IFN-tau* and *PAG2*) suggested that *AGO2*-deficient cells exhibited differentiation but showed several marked cellular and morphological deviation compared to control cells (Figures 5J, 4E and 6). *AGO2*-deficient cells were characterized by reduction (50%) of primary trophoblastic giant cells as well as a significant reduction of their differentiation into secondary cells (especially binuclear cells) compared to control cells (32% vs. 69%; P < 0.05). Overall endopolyploidy level, which is determined by the average number of DAPI stained blue nucleus per cells was found to be significantly reduced (28%) in *AGO2* knocked down cells. Inline with that, a reduced average size of nuclei was observed in the transfected cell. A trophoblast cell was considered apoptotic when showing fragmented nuclei (with blue channel for DAPI) which were significantly higher (42%) in AGO2-siRNA transfected cells compared to non-transfected groups (12%). Likewise, a remarkable reduction in cumulative alkaline phosphatase activity (red signal) in the AGO2-siRNAs transfected cells was observed (Figure 6).

## Discussion

### Differential regulation of miRNAs in day-50 NT and IVP placentas

The aberrant genetic or epigenetic modifications in the NT placentas due to errors in nuclear reprogramming have been evidenced by aberrant non-coding RNA expression, imprinting problems, changes in DNA methylation and histone/chromatin modifications [6,7,10]. Among the non-coding RNAs, miRNAs were found to be aberrantly reprogrammed in cloned mouse blastocyst [13] and bovine day-17 elongated embryos [16]. In the present study a large number of miRNAs were found to be transcriptionally not reprogrammed correctly in the NT (68% miRNA) and IVP (36% miRNA) derived placentas. Relative expression analysis revealed a massive down regulation of miRNAs in day-50 NT and IVP placentas compared to the day-50 placentas from AI pregnancies. This difference could be due to the cumulative effects of somatic cell nuclear transfer and in vitro culture of the resulting embryos. At least 62% of the miRNA studied in day-50 NT placentas were differentially expressed compared to IVP placentas, where 90% of them were found to be downregulated. Overall 36% of the 377 miRNAs studied were differentially regulated between IVP and AI placentas, of which about 96% were downregulated in IVP placentas. These massive deregulations could be entailed to the reported radically altered gene expression in cloned placenta [17,18] associated to poor placentome development in NT pregnancies in the first trimester leading to subsequent pregnancy loss.

Reprogramming errors in NT or IVP derived embryos have been postulated to happen as a multi-step process and the effect could vary depending on the stage of development [19]. It has been suggested that the commonly observed low developmental efficiency of NT embryos may not be largely due to nuclear reprogramming during early embryo development but potentially caused by abnormal gene reprogramming during postimplantation feto-placental development [20]. While, NT blastocyst more closely resemble the in vivo derived ones compared to in vitro fertilized embryos in terms of global gene expression [20], trophoblast lineage was found to be significantly affected by reprogramming errors arising from epigenetic modifications during post blastocyst stage [19]. The results of the present study showed that the major deregulation of miRNAs in NT placentas to be most profound at day 50 of pregnancy. Moreover, differences in their expression are also evident and advancing to expanded blastocyst and embryo elongation stage. In addition, trophectoderm specific miRNAs were found to be aberrantly expressed in the NT and IVP expanded blastocysts compared to that of AI, while inner cell mass specific miRNAs were less or not aberrantly expressed in either NT or IVP expanded blastocysts compared to their AI counterparts.

Those miRNAs, which are located within or nearby imprinted regions of the chromosome, showed a clear deregulation of their expression in NT and IVP expanded blastocyst when compared to expanded AI blastocysts. Collectively, the results of the present study evidenced trophoblast specific and imprinted miRNAs to be aberrantly expressed in NT blastocysts when compared to miRNAs which are specific for the inner cell mass or embryonic stem cells. Similarly, previous studies have reported changes in miRNA expression during trophectoderm specification [21] and aberrant epigenetic reprogramming and thus expression of imprinted miRNAs (e.g. miR-127, miR-136) in cloned mouse embryos [13]. Therefore, a profound deregulation of miRNA expression due to reprogramming failure is found to occur around day 50 of NT pregnancies at which time complex placentomes are developing. The deregulation of miRNAs at the elongated stage could be attributed to the cell specific aberrant expression, but this could not be elucidated as because, elongated embryos were comprised of embryonic and extra-embryonic tissues. However, at day 50 of pregnancy differences with respect to miRNA expression were significantly evident by down regulation of most of them in NT placentas compared to AI counterparts. Interestingly, the degree of miRNA deregulation at day 225 of pregnancy either in NT or IVP compared to AI was found to be very small, indicating temporal regulation of these miRNAs in bovine placentas. At approximately day 50, those fetuses with suboptimal placentome formation and with more deregulated miRNA expression could be slowly starved to death, whereas fetus that progress beyond this stage have better placentome development as studied previously [1].

### Major causes for miRNA deregulation in NT and IVP derived placentas

Major sources of aberrant gene expression in NT placentas have been found to be due to abnormal epigenetic and genetic processes. These include non-coding RNA mediated regulation, aberrant DNA methylation, genomic imprinting and chromatin remodeling. Maintenance of imprinting has been shown to be less dependent on DNA methylation in the placenta compared to the embryo itself, with involvement of repressive histone methylation rather than DNA methylation [9]. The present findings reveal that there is no significant difference in global DNA methylation in elongated day-16 embryos and day-50 placentas derived from NT, IVP and AI. Therefore, it is possible to postulate that other genetic or epigenetic processes including aberrant regional chromatin remodeling rather than global DNA methylation are responsible for massive deregulation of miRNAs in the NT or IVP placentas.

Bioinformatic analysis of the deregulated miRNAs revealed that miRNAs were mostly deregulated as cluster and show similarities in their deregulation between the members within the same miRNA family. They were found to be transcribed in a large transcription units of coding or non-coding genes using their own promoters as an independent entities or as polycistrons [22]. Therefore, any transcriptional or processing disturbance during the earlier stage of cloned embryo and extra embryonic lineage differentiation due to improper reprogramming of specific regulatory molecules responsible for global transcription, processing and maturation of miRNAs could be a potential reason for global down regulation of miRNAs in NT or IVP derived placentas. To elucidate these possibilities, we profiled 18 well known miRNA processing machinery genes in different placentas of different origins. Our results indicate that several of such molecules were not proper reprogrammed in NT or IVP derived placentas when compared to that of AI derived placentas or donor fibroblast cells. Among them, eukaryotic transcription initiation factor 2C2 (*EIF2C2*) or *AGO2* was found to be down regulated in the NT and IVP placentas due to hypermethylation of their promoter region compared to that placentas derived from AI, which could be linked to down regulation of miRNAs as reported previously [23,24]. Expression of *AGO2* has been shown to be stable in trophoblast [25] and important in many biological processes and development including mouse oogenesis [26], early development [27] and maternal-zygotic transition [28]. Embryonic fibroblasts and hematopoietic cells from AGO2 knockout mouse showed reduction of all mature miRNAs [29,30]. Therefore, according to previous reports and findings of this study, it is possible to speculate that global down regulation of miRNAs in the NT and IVP derived day-50 placentas compared to that of AI might be due to deregulation of miRNA processing genes along with aberrant reprogramming of other factors.

According to the results published so far on the role of miRNAs in early mammalian development, it is clear that earliest differentiation events leading to epiblast and trophectoderm lineages proceed normally in the absence of canonical miRNAs [31]. The present study however shows that the second lineage differentiation is affected by deregulation of miRNAs. It has been reported that AGO2-mutant embryos show abnormalities in implanted decidua and hypotrophic placentas including marked reduction in the thickness of the labyrinthine layer [32]. The present study, however, adds many additional cellular and morphological defects in the trophoblast differentiation in vitro including reduced trophoblastic giant cells, endopolyploidy, increased apoptosis and reduced activity of alkaline phosphatase.

## Conclusion

The results of the present study revealed a massive deregulation of miRNAs in bovine day-50 placentas derived from transfer of cloned or in vitro produced bovine embryos. Most of the miRNAs were found to be poorly reprogrammed and to be affected as large chromosomal cluster and miRNA families. These findings suggest that genome wide aberrant expression of miRNAs due to reprogramming failure of miRNA processing regulatory molecules (namely: aberrant AGO2 expression), regional chromatin remodeling affecting polycystronic miRNA expression and imprinting problems in placentas of nuclear transfer derived pregnancy, may result in abnormal regulation of transcriptional activities leading to subsequent pregnancy loss. Taken together, aberrant expression of transcriptional regulatory miRNA molecules during redifferentiation for placentogenesis leading to abnormal genetic and epigenetic modification in the placentas from cloned conceptuses are likely caused by re-programming failure as a consequence of somatic cell nuclear transfer as well as environmental conditions during in vitro culture. Being important gene regulators, miRNAs could display an interesting avenue to resolve numerous open questions in terms of regulatory mechanisms involved in genetic and/or epigenetic aberrations in placentas of SCNT or IVP pregnancies.

## Methods

### In vitro embryo production (IVP) and processing of blastocysts

Bovine ovaries were collected from local abattoir and cumulus-oocyte complexes (COCs) were aspirated from 2- to 8-mm-diameter follicles. COCs with multiple cumulus layers including evenly granulated cytoplasm were selected, matured and fertilized in vitro using the frozen semen of one single bull and developing embryos were subsequently cultured in vitro using a standard protocol. At day 7 (blastocyst) and day 8 (fully expanded blastocyst) of embryo culture, a total of 5–8 embryos (washed in PBS) were freezed in liquid nitrogen. A total of 20 embryos were fixed in 4% parafomaldehyde overnight at 4°C for whole mount in situ hybridization. Additionally, in vitro derived day-7 blastocysts (n = 20) were transferred singly to the recipients to generate day-16, day-50 and day-225 pregnancies (details of the procedure are described in Additional file 1).

### In vivo embryo collection and generation of control pregnancies (artificial insemination)

Simmental heifers were synchronized by intra muscular injection of cloprostenol (PGF_2_α, Estrumate; Essex Tierarznei, Munich, Germany) twice within 11 days and subsequently superovulation was performed by injection of FSH (Stimufol, Ulg FMV, Belgium) starting at day 11 after onset of estrus. Frozen–thawed semen was used to inseminate all heifers. The blastocysts were flushed out with 500 ml D-PBS at day 7.5 post inseminations. Only morphologically good-quality early blastocyst and expended blastocysts (5 embryos per pool in triplicate for both stages) were snap frozen. Moreover, a total of 20 expanded blastocysts were fixed as mentioned before for in situ hybridization. Heifers (n = 15) synchronized with a single dose of PGF_2_α followed by estrus check were artificially inseminated 10 hours after standing estrus using frozen semen of the same sire used for production of IVP embryos. Pregnancies were terminated at days 16, 50 and 225 to collect elongated day-16 control embryos as well as placenta tissues at day 50 (n = 3) and day 225 (n = 4) of pregnancies. All animal were handled according to animal protection law of Germany. Moreover, the experiments conducted in this study are approved by the Animal Protection Chair of the University of Bonn.

### Donor cell preparation and nuclear transfer

Preparation of donor cells, nuclear transfer and embryos culture has been performed according to the protocol described elsewhere with some modification (Additional file 1) [33]. Triplicate (5 in each) of both early and expanded blastocysts were frozen as described above. Additionally, blastocysts derived from NT were fixed for whole mount in situ hybridization and a total of 30 blastocysts was transferred singly to synchronized recipients (n = 30).

### Recipient preparation and embryo transfer

Estrous synchronization and transfer of embryos to the recipients has been carried out according to the previous report [34]. Single NT (n = 30) and IVP (n = 20) derived embryos that were of good or excellent quality (grades 1 or 2) were transferred into the uterine horn ipsilateral to the corpus luteum of recipients, respectively (details in the Additional file 1: supplementary methods).

### Pregnancy monitoring and retrieval of experimental material

Recipients were monitored for coming back to estrus at day 21 to be considered as non-pregnant. Pregnancy diagnosis was performed at days 28 and 42 of gestation by transrectal ultrasonography and by rectal palpation at day 42 and 56. Recipients being pregnant after transfer of IVP or NT derived embryos as well as heifers being pregnant after AI were slaughtered gradually at day 16 (IVP-N = 5, AI-N = 5, NT-N = 5), day 50 (IVP-N = 3, AI-N = 3, NT-N = 3) and day 225 (IVP-N = 4, AI-N = 4, NT-N = 4) of pregnancy. Elongated embryos at day 16, chorioallantois with early cotyledons at day 50 and placentomes (fetal cotyledonary tissues) at day 225 of pregnancy were collected, washed twice in PBS and stored in RNA later (Ambion Inc, TX, USA).

### Extraction and purification of small RNAs from placentas

Total RNAs from the three individual frozen placentas (15 mg) of each group of pregnancy (IVP, NT and AI) and fibroblast cells (4 × 10^6^) were isolated using miRNeasy mini kit (QIAGEN GmbH, Hilden, Germany). Large (>200 nt) and small RNAs (<200 nt) were separated using RT^2^ qPCR-Grade miRNA isolation kit (SABioscienecs, Frederick, MD, USA) according to manufacturer’s instructions. The quality and the concentration of the small and large RNAs were assessed by NanoDrop 8000 spectrophotometer (NanoDrop, Wilmington, Delaware, USA).

### Genomic DNA, total RNA and protein extraction

Whole individual elongated embryos at day 16, placentas at day 50 (15 mg) and placentomes at day 225 (15 mg) of pregnancy from IVP, NT and AI (at least three of each) were used for isolation of gDNA, total RNA and protein using DNA/RNA/Protein purification kit (Norgen Biotek corporation, Thorold, Canada) according to the methods recommended by the manufacturer. In addition, 5 early blastocysts and 5 expanded blastocysts (from each IVP, NT and AI method) were also used to isolate total RNAs using the same procedures. The quality and the concentration were assessed by NanoDrop 8000 spectrophotometer (NanoDrop, Wilmington, Delaware, USA).

### Large scale expression profiling of miRNAs by real-time quantitative PCR

Total of 166 ng small RNAs from 3 placentas at day 50 derived from every group of pregnancy (IVP, NT and AI) and donor cells (in triplicate) were synthesized into first strand cDNAs using RT^2^ miRNA first strand kit (SABiosciences). Real time qPCR of miRNAs was performed using 384-well miRNAs primed PCR plate (SABiosciences) comprised of 377 individual miRNAs (most of them are conserved in human, mouse and bovine), 4 endogenous controls (U6, Snord44, Snord47 and Snord48), 2 reverse transcription controls and 2 positive PCR controls according to the protocols provided by the manufacturer. The assays were performed in ABI 7900 HT real time PCR system (Applied Biosystems, Foster City, CA, USA) with sybr green technology (SABiosciences). Data were analysed by ΔΔC_t_ method and normalization was performed by geometric mean of four endogenous controls through SAbiosciences’s PCR array data analysis on-line web-based analysis portal provided with t test. A fold regulation 2 or more with the value of P less than/equal to 0.05 were considered as significant different expression.

### Whole mount blastocyst in situ hybridization of miRNAs

Whole mount in situ hybridization of miRNAs in the in vitro blastocysts was performed as described elsewhere [35]. At least three embryos were used for the hybridization of each miRNA. According to the expression patterns miRNAs in IVP embryos, selected candidate trophoblast and inner cell mass specific and imprinted miRNAs were localized to the expanded AI and NT blastocysts. Embryos were mounted individually with VectaShield containing DAPI (Vector laboratories, Burlingame, CA) and analyzed by confocal laser scanning microscope (CLSM LSM-510, Carl Zeiss, Germany). Scramble negative controls probes have been used to prove the specificity of hybridization of miRNA probes.

### Reverse transcription and SYBR green qPCR for selected miRNAs

Expression of selected miRNAs has been examined in blastocyst, expanded blastocyst, day-16 elongated embryo, day-50 placenta and day-225 placentome (from AI, IVP and SCNT). All the reagents and kits used for this purpose were obtained from Exiqon (Exiqon, Vedbaek, Denmark). A 36 ng total RNA from each sample was applied to synthesize first strand cDNA using Universal cDNA synthesis kit. Real time qPCR was performed using LNA™ PCR primer set for mir- 21, -24, -127-3p, -135b, -299-5p, -302, -376a, -431, and mir-544a with universal RT primers using SYBR Green master mix in ABI PRISM® 7000 sequence detection system (Applied Biosystems, Foster City, CA, USA). Dilution of cDNA, preparation of mix and thermal cycling condition was performed as recommended by the manufacturer. Data were analyzed as mentioned before except, normalization was performed using the mean Ct value of U6 RNA and 5S ribosomal RNA.

### Reverse transcription and SYBR green qPCR for miRNA processing genes

Important candidate genes involved in transcription, processing and generating mature miRNAs were quantified in day-50 placentas derived from IVP, AI and NT pregnancy by qPCR (Additional file 1: supplementary methods). Reverse transcription of 600 ng total RNA from each sample was performed using Superscript II Reverse Transcriptase (Invitrogen, Carlsbad, CA) in combination with random primers (Invitrogen, Carlsbad, CA) and oligo (DT)_23_ (Sigma). All primers utilized were designed and optimized in order to ensure optimum reaction efficiencies both for target and housekeeping reference genes (GAPDH, Histone). Triplicate reactions were performed for each gene by standard PCR protocol recommended for the instrument. Data were normalized and subsequent analysis was performed using ΔΔCt methods [36] and analyzed as mentioned before (details in the Additional file 1).

### Quantification of global DNA methylation

Genomic DNA from the 3 elongated day-16 embryos and 3 day-50 placentas from each IVP, AI and SCNT pregnancy was used to quantify the global methylation status using Methylamp Global DNA Methylation Quantification Ultra kit (Epigentek, Brooklyn, NY) according to user instruction. Briefly, 200 ng of genomic DNA from each sample was immobilized to the strip well and methylated fraction of DNA was recognized by 5-methylcytosine antibody. Color was developed and absorbance reading was performed in ThermoMax microplate reader (Molecular Devices, Sunnyvale, CA) at 450 nm and subsequent analysis was performed according to the user instruction of Methylamp Global DNA Methylation Quantification Ultra kit.

### Bisulfite genomic sequencing

Putative promoter region of bovine AGO2/EIF2C2 (chromosome 14: 2372494–2373020, sense strand) 500 bases upstream to the first exon has been used to design primers for the amplification of bisulfite converted DNA using MethPrimer and Methyl Primer Express® Software v1.0 (Applied biosystem, Foster City, CA). About 500 ng genomic DNA was used for bisulfite conversion using EZ DNA Methylation Kit (Zymo Research, Orange, CA) according to manufacturers’ instruction. Each converted DNA upon amplifying the putative promoter region of AGO2 were purified using QIAquick PCR purification kit (QIAGEN GmbH, Hilden, Germany) and then cloned into the pGEM-T Easy vector (Promega, Mannheim, Germany) and transform into *E. Coli*. A minimum of 10 different clones from every sample were randomly selected and processed for sequencing with M13 primers using CEQ8000 sequencer system (Beckman Coulter, Brea, CA). Conversion efficiency and methylation sites were analyzed by Quantification Tool for Methylation Analysis [37] (details in Additional file 1).

### Western blotting

Total proteins from 3 day-50 placentas from same group (IVP, AI and SCNT) were pooled equally (30 μg) and separated by SDS-PAGE (gradient 4-18%) and transferred onto a nitrocellulose membrane (Amersham Biosciences). Blocking was performed in buffer (20 mM Tris pH 7.5, 150 mM NaCl, 0.05% Tween-20 and 1% polyvinylpyrolidone) at room temperature for 1 hour and then incubated with goat anti-eIF2C2 polyclonal antibody **(**Santa Cruz Biotechnology, Santa Cruz, CA) overnight at 4°C. The membrane was incubated for 1 h at room temperature with secondary antibody (HRP-conjugated mouse anti-goat IgG). The chemiluminescence was detected using ECL plus western blotting detection system (Amersham Biosciences) and visualized by using Kodak BioMax XAR film. The membrane was stripped by incubation in 2% SDS, 100 mM Tris–HCl and 0.1% beta-mercaptoethanol for 30 min at 60°C and re-probed with GAPDH antibody (loading control).

### Derivation and culture of bovine TBSs

Bovine blastocysts were produced in vitro by the similar method used for production of embryos which were transferred to the recipient cows for the generation of IVP pregnancies as mentioned earlier. TBSs from in vitro produced blastocysts were derived according to the method described elsewhere [38]. Briefly, on day 9, hatched blastocysts were individually cultured onto collagen-coated dishes in the uterine fibroblast conditioned medium [Dulbecco’s modified Eagle’s/F-12 medium (Gibco) containing 100 IU/ml penicillin and 100 μg/ml streptomycin (Sigma), supplemented with 50% bovine uterine fibroblast conditioned medium, 10% FBS, 50 μm β-mercaptoethanol] at 37°C in an atmosphere of 5% CO2. Blastocysts were found to be attached within a few days having remarkable cell outgrowth and proliferation (Figures 5A,B and C). The medium was changed every 2 or 3 days and mechanically passaged the confluent cell sheets by pipetting, which were plated onto collagen-coated 25-cm^2^ flasks (Figure 5D) and cultured till to become confluent (Figure 5F). Cells were maintained for at least 20 passages without any apparent changes in their morphology and viability.

### TBSs differentiation in vitro and AGO2 knock down

Established bovine trophoblastic cells were differentiated in vitro on collagen substrata according to the method developed earlier [39] with some modification. Briefly, mechanically dissociated cell sheets at passage 18–20 (denoted as day 1) were to broken down into very small cell clumps through vigorous pipetting using 1000 μl tips, were plated onto collagen-mounted 25-cm^2^ flask in differentiation medium [Dulbecco’s modified Eagle’s/F-12 medium containing 100 IU/ml penicillin and 100 μg/ml streptomycin (Sigma), supplemented with 10% FBS, 50 μm β-mercaptoethanol] for differentiation. The medium was changed 3 times during 12 day culture period in 3 days interval, where amount of FBS was reduced by 3% from the differentiation medium each time to reach 1% at the end. Differentiation was confirmed by the presence of four distinct terminal cells namely trophoblast giant cells, spongiotrophoblast, glycogen cells, and syncytial trophoblast like cells.

Two FlexiTube siRNAs for bovine *AGO2* targeting two different position of mRNA sequence CAGGTTCTGCACCACGAGTTA (accession no. NM_205794.1, 2039–2060) and CCGAGGAGAGTTAACAGGGAA (accession no. NM_205794.1, 206–227) with the modification of 3´Alexa Fluor 488 were designed and synthesized at 20 nM by Qiagen (QIAGEN GmbH, Hilden, Germany). Dissociated and homogenous cells from each culture flask divided in to 3 where one for transfection of AGO2 siRNA, one for scrambled siRNA and third one for only transfection reagents without any siRNAs as negative control. Trophoblast cell (2 × 10^4^ cells/ml) during culture for differentiation on 2nd day were transfected with of the siRNA duplex at a final concentration of 5 nM using HiPerFect Transfection Reagent (QIAGEN GmbH, Hilden, Germany) in 24-well plates (mounted with collagen gel) according to the manufacturer’s instructions. Cells were incubated with the complexes for 24 hours and medium was changed by fresh differentiation media having 7% FBS. Cells were transfected again with siRNAs transfection complex on day 5. Similar to siRNA, a separate group of cells were transfected with non-specific scrambled siRNAs and only transfection regents for another group were performed. Cells were dissociated through standard trypsinization and washed three times in cold PBS. All results represent experiments conducted in triplicate at least two times. Similar to well plate, three separate cultures and transfections of cell were performed in CoverWell perfusion chambers (Science Services GmbH, Muenchen, Germany, Cat# E70326-46) attached with microscope slide which were mounted with collagen gel exactly like flasks prepared prior to differentiation culture. All the transfection resulted in uptake of the siRNAs in more than 90% of the cells as assessed by green fluorescein signal by light fluorescent microscope. Analyses of the effects of miRNAs on cell development were performed 6–7 days after transfections.

### Characterization of transfected trophoblast cells and immuno-histochemistry

Cells were dissociated through standard trypsinization and washed three times in cold PBS. Triplicate samples from each treatment in 24 well plates of first time experiment were used to isolate large RNA to quantify the expression of *AGO2* mRNA, small RNA to quantify the expression of selected miRNAs and proteins to analyze the changes of AGO2 protein from the same cells according to the method mentioned earlier in case of placentas. Cells from parallel batch were used to analyze proliferation and viability using standard trypan blue staining followed by counting in heamocytometer under light microscope. Cells knocked down of *AGO2* and differentiated in perfusion chambers were used for immuno-histochemistry (for detection of AGO2 expression) together with detection of alkaline phosphatase activity (APA), status of differentiation into terminal cells and degree of endoreduplication in the same chamber slide. For immuno-histochemistry, chamber slides were first fixed in 4% paraformaldehyde at 4°C for 10 minutes and washed 3 times in PBS. Refixation was carried out with cold (-20°C) acetone for 5 minutes followed by 3 times washing in PBS and subsequent with PBS containing 1% BSA. After blocking the slides were incubated with AGO2 polyclonal antibody in blocking solution (1:100) overnight at 4°C followed by washing 3 times in PBS and incubation with anti-goat donkey antibody conjugated with Alexa Fluor® 488 (Invitrogen, Carlsbad, CA) (1:200) for 1 hour at room temperature. Slides were washed 3 times in PBS and equilibrated in 1 M tris pH 8.2 for 10 minutes and 2 drops of fast red solution (Fast Red Substrate System, Cat# K0699, Dako, Glostrup, Denmark) were added to each chamber for staining the APA for 30 minutes at RT. Antibody diluent was used in the experiment as negative control. After washing the chambers 3 times in PBST, perfusion chamber were released from the glass slide. Slides were mounted with VectaShield containing DAPI (Vector laboratories, Burlingame, CA) and analyzed by confocal laser scanning microscope (CLSM LSM-510, Carl Zeiss, Germany). Green, Cy5 and DAPI filter was used to detect AGO2 expression, APA and nuclear status including endoreduplication, respectively. Over 500 cells from at least 9 images were analyzed per experiment. Irrelevant IgG was assessed to be negative.

## Competing interests

The authors declare that they have no conflict of interest in relation to this work.

## Authors’ contributions

MMH was responsible for conducting the experiment, analysis, interpretation of data and drafting the manuscript. DT has made substantial contributions to conception, design and has contributed as the corresponding author. DSW participated in its design and coordination and helped to draft the manuscript. MJP has contributed to the expression analysis of miRNAs processing machinery genes. KS contributed by supervising the work with necessary suggestion and revising it critically for important intellectual content. MH, EH and FR have contributed by helping the preparation of biological samples. MH was responsible for project development and has made substantial contributions to conception. GK contributed to microscopic image of IHC staining. CL, ET and JU have contributed to the design and reading of the draft manuscript during prepration. All contributing authors have made substantive intellectual contributions to this work, reviewed the manuscript and approved before submitting the final copy of this manuscript.

## Supplementary Material

Additional file 1 **Is available with the online version of this paper which contains detailed experimental procedures, result of primary analysis as graph (Figure S1, S2 and S3), a table (Table S1) listing the primers and oligos used in the experiment.** Click here for file

## References

[B1] Hill JR Burghardt RC Jones K Long CR Looney CR Shin T Spencer TE Thompson JA Winger QA Westhusin ME Evidence for placental abnormality as the major cause of mortality in first-trimester somatic cell cloned bovine fetuses Biol Reprod 2000 15 6 1787 1794 10.1095/biolreprod63.6.1787 11090450

[B2] Yang X Smith SL Tian XC Lewin HA Renard JP Wakayama T Nuclear reprogramming of cloned embryos and its implications for therapeutic cloning Nat Genet 2007 15 3 295 302 10.1038/ng1973 17325680

[B3] Smith LC Murphy BD Genetic and epigenetic aspects of cloning and potential effects on offspring of cloned mammals Cloning Stem Cells 2004 15 2 126 132 10.1089/1536230041372319 15268786

[B4] Tamada H Kikyo N Nuclear reprogramming in mammalian somatic cell nuclear cloning Cytogenet Genome Res 2004 15 2–4 285 291 15237217 10.1159/000078200 PMC2078605

[B5] Niemann H Tian XC King WA Lee RS Epigenetic reprogramming in embryonic and foetal development upon somatic cell nuclear transfer cloning Reproduction 2008 15 2 151 163 10.1530/REP-07-0397 18239046

[B6] Holmes R Soloway PD Regulation of imprinted DNA methylation Cytogenet Genome Res 2006 15 1–4 122 129 16575171 10.1159/000090823

[B7] Li E Beard C Jaenisch R Role for DNA methylation in genomic imprinting Nature 1993 15 6453 362 365 10.1038/366362a0 8247133

[B8] Oudejans C Westerman B van Elk E Konst A Mulders M Alders M van Vugt J van Wijk I Mannens M Growth regulation of extraembryonic tissues. The effect of genomic imprinting on development of the placenta Eur J Obstet Gynecol Reprod Biol 1997 15 1 29 32 10.1016/S0301-2115(97)00195-4 9447343

[B9] Wagschal A Feil R Genomic imprinting in the placenta Cytogenet Genome Res 2006 15 1–4 90 98 16575167 10.1159/000090819

[B10] Lewis A Mitsuya K Umlauf D Smith P Dean W Walter J Higgins M Feil R Reik W Imprinting on distal chromosome 7 in the placenta involves repressive histone methylation independent of DNA methylation Nat Genet 2004 15 12 1291 1295 10.1038/ng1468 15516931

[B11] Ambros V The functions of animal microRNAs Nature 2004 15 7006 350 355 10.1038/nature02871 15372042

[B12] Kloosterman WP Plasterk RH The diverse functions of microRNAs in animal development and disease Dev Cell 2006 15 4 441 450 10.1016/j.devcel.2006.09.009 17011485

[B13] Cui XS Zhang DX Ko YG Kim NH Aberrant epigenetic reprogramming of imprinted microRNA-127 and Rtl1 in cloned mouse embryos Biochem Biophys Res Commun 2009 15 2 390 394 10.1016/j.bbrc.2008.12.148 19126398

[B14] Kircher M Bock C Paulsen M Structural conservation versus functional divergence of maternally expressed microRNAs in the Dlk1/Gtl2 imprinting region BMC Genomics 2008 15 346 10.1186/1471-2164-9-346 18651963 PMC2500034

[B15] Elsik CG Tellam RL Worley KC Gibbs RA Muzny DM Weinstock GM Adelson DL Eichler EE Elnitski L Guigo R The genome sequence of taurine cattle: a window to ruminant biology and evolution Science 2009 15 5926 522 528 19390049 10.1126/science.1169588 PMC2943200

[B16] Castro FO Sharbati S Rodriguez-Alvarez LL Cox JF Hultschig C Einspanier R MicroRNA expression profiling of elongated cloned and in vitro-fertilized bovine embryos Theriogenology 2010 15 1 71 85 10.1016/j.theriogenology.2009.08.003 19836069

[B17] Aston KI Li GP Hicks BA Sessions BR Davis AP Winger QA Rickords LF Stevens JR White KL Global gene expression analysis of bovine somatic cell nuclear transfer blastocysts and cotyledons Mol Reprod Dev 2009 15 5 471 482 10.1002/mrd.20962 19062181

[B18] Oishi M Gohma H Hashizume K Taniguchi Y Yasue H Takahashi S Yamada T Sasaki Y Early embryonic death-associated changes in genome-wide gene expression profiles in the fetal placenta of the cow carrying somatic nuclear-derived cloned embryo Mol Reprod Dev 2006 15 4 404 409 10.1002/mrd.20345 16435373

[B19] Jouneau A Zhou Q Camus A Brochard V Maulny L Collignon J Renard JP Developmental abnormalities of NT mouse embryos appear early after implantation Development 2006 15 8 1597 1607 10.1242/dev.02317 16556918

[B20] Smith SL Everts RE Tian XC Du F Sung LY Rodriguez-Zas SL Jeong BS Renard JP Lewin HA Yang X Global gene expression profiles reveal significant nuclear reprogramming by the blastocyst stage after cloning Proc Natl Acad Sci U S A 2005 15 49 17582 17587 10.1073/pnas.0508952102 16314565 PMC1308920

[B21] Viswanathan SR Mermel CH Lu J Lu CW Golub TR Daley GQ microRNA expression during trophectoderm specification PLoS One 2009 15 7 e6143 10.1371/journal.pone.0006143 19582159 PMC2702083

[B22] Kim VN MicroRNA biogenesis: coordinated cropping and dicing Nat Rev Mol Cell Biol 2005 15 5 376 385 10.1038/nrm1644 15852042

[B23] Cifuentes D Xue H Taylor DW Patnode H Mishima Y Cheloufi S Ma E Mane S Hannon GJ Lawson ND A novel miRNA processing pathway independent of Dicer requires Argonaute2 catalytic activity Science 2010 15 5986 1694 1698 10.1126/science.1190809 20448148 PMC3093307

[B24] Zhang X Graves PR Zeng Y Stable Argonaute2 overexpression differentially regulates microRNA production Biochim Biophys Acta 2009 15 2 153 159 10.1016/j.bbagrm.2008.11.004 19064005

[B25] Donker RB Mouillet JF Nelson DM Sadovsky Y The expression of Argonaute2 and related microRNA biogenesis proteins in normal and hypoxic trophoblasts Mol Hum Reprod 2007 15 4 273 279 10.1093/molehr/gam006 17327266

[B26] Kaneda M Tang F O'Carroll D Lao K Surani MA Essential role for Argonaute2 protein in mouse oogenesis Epigenetics Chromatin 2009 15 1 9 10.1186/1756-8935-2-9 19664249 PMC2736168

[B27] Morita S Horii T Kimura M Goto Y Ochiya T Hatada I One Argonaute family member, Eif2c2 (Ago2), is essential for development and appears not to be involved in DNA methylation Genomics 2007 15 6 687 696 10.1016/j.ygeno.2007.01.004 17418524

[B28] Lykke-Andersen K Gilchrist MJ Grabarek JB Das P Miska E Zernicka-Goetz M Maternal Argonaute 2 is essential for early mouse development at the maternal-zygotic transition Mol Biol Cell 2008 15 10 4383 4392 10.1091/mbc.E08-02-0219 18701707 PMC2555945

[B29] Diederichs S Haber DA Dual role for argonautes in microRNA processing and posttranscriptional regulation of microRNA expression Cell 2007 15 6 1097 1108 10.1016/j.cell.2007.10.032 18083100

[B30] O'Carroll D Mecklenbrauker I Das PP Santana A Koenig U Enright AJ Miska EA Tarakhovsky A A Slicer-independent role for Argonaute 2 in hematopoiesis and the microRNA pathway Genes Dev 2007 15 16 1999 2004 10.1101/gad.1565607 17626790 PMC1948855

[B31] Suh N Baehner L Moltzahn F Melton C Shenoy A Chen J Blelloch R MicroRNA function is globally suppressed in mouse oocytes and early embryos Curr Biol 2010 15 3 271 277 10.1016/j.cub.2009.12.044 20116247 PMC2872512

[B32] Cheloufi S Dos Santos CO Chong MM Hannon GJ A dicer-independent miRNA biogenesis pathway that requires Ago catalysis Nature 2010 15 7298 584 589 10.1038/nature09092 20424607 PMC2995450

[B33] Hölker M Petersen B Hassel P Kues WA Lemme E Lucas-Hahn A Niemann H Duration of in vitro maturation of recipient oocytes affects blastocyst development of cloned porcine embryos Cloning Stem Cells 2005 15 1 35 44 10.1089/clo.2005.7.35 15996116

[B34] El-Sayed A Hoelker M Rings F Salilew D Jennen D Tholen E Sirard MA Schellander K Tesfaye D Large-scale transcriptional analysis of bovine embryo biopsies in relation to pregnancy success after transfer to recipients Physiol Genomics 2006 15 1 84 96 10.1152/physiolgenomics.00111.2006 17018689

[B35] Hossain MM Ghanem N Hoelker M Rings F Phatsara C Tholen E Schellander K Tesfaye D Identification and characterization of miRNAs expressed in the bovine ovary BMC Genomics 2009 15 443 10.1186/1471-2164-10-443 19765282 PMC2762473

[B36] Livak KJ Schmittgen TD Analysis of relative gene expression data using real-time quantitative PCR and the 2(-Delta Delta C(T)) Method Methods 2001 15 4 402 408 10.1006/meth.2001.1262 11846609

[B37] Kumaki Y Oda M Okano M QUMA: quantification tool for methylation analysis Nucleic Acids Res 2008 15 Web Server issue W170 W175 18487274 10.1093/nar/gkn294 PMC2447804

[B38] Shimada A Nakano H Takahashi T Imai K Hashizume K Isolation and characterization of a bovine blastocyst-derived trophoblastic cell line, BT-1: development of a culture system in the absence of feeder cell Placenta 2001 15 7 652 662 10.1053/plac.2001.0702 11504534

[B39] Nakano H Shimada A Imai K Takezawa T Takahashi T Hashizume K Bovine trophoblastic cell differentiation on collagen substrata: formation of binucleate cells expressing placental lactogen Cell Tissue Res 2002 15 2 225 235 10.1007/s00441-001-0491-x 11845329

