# Supplementary material for: Massive deregulation of miRNAs from nuclear reprogramming errors during trophoblast differentiation for placentogenesis in cloned pregnancy
Source: BMC Genomics. 2014 Jan 18;15:43. doi: 10.1186/1471-2164-15-43 (PMC3904697; doi:10.1186/1471-2164-15-43)
Supplement: Additional file 1 — Is available with the online version of this paper which contains detailed experimental procedures, result of primary analysis as graph (Figure S1, S2 and S3), a table (Table S1) listing the primers and oligos used in the experiment. [file 1471-2164-15-43-S1.doc]

**Supplementary Materials**

**In vitro production (IVP) and processing of blastocysts**

Bovine ovaries were collected from local abattoir and transported to the laboratory in a thermosflask (35°C) containing physiological NaCl solution (0.9% NaCl supplemented with 50 μl/100 ml Streptocombin (Albrecht GmbH, Germany). Cumulus-oocyte complexes (COCs) were aspirated from 2- to 8-mm-diameter follicles using a 10-ml syringe loaded with an 18- gauge needle upon washing the ovaries once with 70% ethanol and twice with physiological saline. Quality of COCs has been assessed under stereomicroscope and those with multiple cumulus layers including evenly granulated cytoplasm were selected, washed three times in pre-warmed maturation (MPM supplemented with 12 % heat-inactivated estrous cow serum, 10 μg/ml FSH, 0.73 mg/ml of sodium bicarbonate, 50 μg/ml of gentamicin, 0.23 mg/ml of sodium pyruvate, 1.27 mg/ml HEPES and 0.55 mg/ml calcium lactate). Selected COCs were subsequently transferred in groups of 50 to each well of four-well dishes (Nunc, Roskilde, Denmark) containing 400 μ1 maturation medium without being covered with mineral oil. Maturation was carried out at 39°C in a humidified atmosphere with 5% CO_2_ for 22 h. Sperm cells were separated by ‘‘swim up’’ technique for in vitro fertilization (IVF) according to (Parrish et al. 1988), where 50 matured oocytes in a well were co-cultured for 18 hours with 1 X 10^6^ spermatozoa/ml at the same condition followed for maturation step. Following IVF, presumed zygotes were gently vortexed to separate them from the surrounding cumulus cells and attached or dead spermatozoa. Cumulus free zygotes were washed three times in CR-1aa culture medium (Rosenkrans & First 1994) supplemented with 10% oestrus cow serum, 10 μl/ml basal medium Eagle (BME-essential amino acids) and 10 μl/ml minimum essential medium (MEM-non essential amino acids) and were transferred into the well containing 400 μl culture medium covered with mineral oil. Embryos were cultured in vitro for 8 days. At day 7 (blastocyst) and day 8 (fully expanded blastocyst) of culture, 5-8 embryos (washed in PBS) were freezed in liquid nitrogen in triplicates with 2 μl of lysis buffer [(5mM DTT, Promega, P1171), 0.8% Igepal (Sigma, I 3021), 1U/ μl RNasin (Promega, N 2511)]. A group of 20 embryos from each developmental stage were fixed in 4% parafomaldehyde overnight at 4°C for whole mount in situ hybridization. In addition, in vitro derived day-7 blastocysts (n=20) were transferred singly to the recipients by nonsurgical standard procedures to generate day-16, day-50 and day-225 pregnancies.

**In vivo embryo production and establishing control (artificial insemination) pregnancy**

To collect in vivo derived blastocysts, six Simmental heifers were synchronized by intra muscular injection of cloprostenol (PGF_2_α, Estrumate; Essex Tierarznei, Munich, Germany) twice within 11 days and subsequently superovulation was performed by injection of FSH (Stimufol, Ulg FMV, Belgium) starting at day 11 after onset of estrus. Frozen–thawed semen was used to inseminate all heifers. The blastocysts were flushed out with 500 ml D-PBS at day 7.5 post inseminations by embryo flushing catheter (CH15, Wörrlein, Ansbach, Germany) fixed in the uterine horn. All flushed blastocysts were assessed under stereo microscope for their quality and stages. Only morphologically good-quality early blastocyst and expended blastocysts (5 embryos per pool in triplicate for both stages) were snap frozen for the isolation of RNA as mentioned before. Another group of 20 expanded blastocysts were fixed overnight in 4% paraformaldehyde at 4°C for whole mount in situ hybridization. Heifers (n=15) synchronized with a single dose of PGF_2_α followed by estrus check were artificially inseminated (AI) using frozen semen of the same sire (except the sire used to generate day 225 pregnancies) after 10 hours of standing estrus and pregnancy were maintained to provide control placenta tissue at day-50 (n=3) and day-225 (n=4).

**Donor cell preparation and nuclear transfer**

Preparation of donor cells, nuclear transfer and culture of reconstructed embryos has been performed according to the protocol described elsewhere with some modification [1]. Briefly, a primary cell line was established from ear skin biopsy of the bull used also production of in vivo and in vitro derived embryos and placentas. The biopsy was minced, washed, dispersed in T25 cell culture flask and cultured in Dulbecco’s Modified Eagles Medium (DMEM) supplemented with 2 mM glutamine, 1% non-essential amino acids, 0.1 mM β-mercaptoethanol, 100 U/ml penicillin, 100 µg/ml streptomycin (all from Sigma, Deisenhofen, Germany) containing 10% fetal calf serum (FCS) (Gibco, Karlsruhe, Germany) in a humidified atmosphere of 95% air and 5% CO_2_ at 37°C. Outgrowing cells were trypsinized (0.05% Trypsin/0.53 mM EDTA; Gibco) and replated to allow proliferation to 90% confluence. The harvested cells were reconstituted at a concentration of 1x 10^6^ cells/ml and then either frozen in 10% dimethyl sulfoxide (Sigma, Deisenhofen, Germany) in DMEM until later use or returned to culture. The fibroblasts used for nuclear transfer were from passages 4-5 and were induced to enter a period of quiescence (presumptive G0) by serum starvation for 7 days (0.5% fetal calf serum).

Oocytes were placed in hepes-buffered TCM-199 (25mM Hepes, 5mM NaHCO_3_) medium containing 1 µg/ml Hoechst 33342 (Sigma, B-2261) and 7.5 µg/ml cytochalasin B (Sigma, C-6762) for ~ 10 min prior to enucleation. Metaphase II oocytes were enucleated by removal of the polar body and the attached cytoplasm with the metaphase plate utilizing a 25 µm beveled glass pipette under the microscope. The absence of the metaphase plate or enucleation was confirmed by a brief exposure of the karyoplast to ultraviolet light. Successful enucleation was also indicated by the typical blue fluorescence of Hoechst 33342 within the pipette. Cytoplasts derived from enucleated oocytes were maintained in TCM-air for up to 2 h and nuclear transfer was conducted in the same medium as enucleation, but without Hoechst 33342 stain. Immediately before donor cell transfer, a suspension of the donor cells was prepared by standard trypsinization. The cells were pelleted and resuspended in TCM-air and remained in this medium until injection. A single cell was sucked into a 30-µm (outer diameter) bevelled glass pipette and carefully transferred into the perivitelline space of the recipient oocyte in close contact with the oocyte membrane. Reconstructed embryos were electrically fused at 26 h after onset of maturation. Fusion of donor cell and oocyte was induced with a single electrical pulse of 25 V DC for 45 µsec between two electrodes with a spacing of 150 µm by electrofusion machine (CFA 400; Kruess, Hamburg, Germany). Fusion was assessed approximately 45 min later by light microscopy. At 28-29 hours after onset of maturation, the reconstructed embryos were chemically activated by incubation in 5 µM ionomycin (Sigma) in hepes-buffered TCM 199 for 4 min followed by a 3.5 hours incubation in 2 mM 6-dimethylaminopyridine (6-DMAP; Sigma) in CR-1aa culture medium at 39°C.

In vitro culture of activated reconstructed complexes has been performed as described for the production of in vitro blastocyst. Developmental rates were assessed periodically up to day 7.5 of culture. Produced embryos were used for three purposes in this study. First, 5 triplets of both early and expanded blastocysts in were freezed as described before. In addition, another group of embryos were fixed overnight in 4% paraformaldehyde for whole mount in situ hybridization. Finally, rest of the blastocysts were transferred singly to the synchronous recipients (n=30) by nonsurgical standard procedures as described below.

**Recipient preparation and embryo transfer**

Estrous synchronization and transfer of embryos to the recipients has been carried out according to the previous report [2]. Briefly, estrous cycles in normal cycling heifers were synchronized by intra-muscular injection of prostaglandin F2a (2 ml Estrumate; Fa. Essex, Germany) followed by a second administration 11 days later. Standing estrus was monitored and embryos were matched with synchronous recipients of no more than ~12 h asynchrony. Single NT (n=30) and IVP (n = 20) embryos that were of good or excellent quality (Grades 1 or 2) were transferred into the uterine horn ipsilateral to the corpus luteum of recipients, respectively.

**Pregnancy monitoring and retrieval of experimental material**

All recipients were monitored for coming back to estrus at day 21. Heifers that returned to estrus at day 21 were considered as non-pregnant. Pregnancy diagnosis was performed at gestation days 28 and 42 by transrectal ultrasonography (Pie Medical, 5 MHz) and by rectal palpation at day 42 and 56. A viable pregnancy was defined as the presence of fetus with a detectable heartbeat. Beginning on Day 120, recipients underwent repeated transabdominal ultrasonography (Pie Medical, 3,5 MHz) every 2–3 wk until day 220. Recipients of IVP embryos, NT embryos and artificial insemination were slaughtered at day 16 (IVP-N=5, AI-N=5, NT-N=5), day 50 (IVP-N=3, AI-N=3, NT-N=3) and day 225 (IVP-N=4, AI-N=4, NT-N=4) of pregnancy. Assessment of the morphometric quality or any abnormalities in the embryos, fetus and placenta were noted accordingly. On day 16, the entire conceptus was weighed together with measurement of length; on Day 50 the fetus and placenta were weighed separately; on Day 225 the fetus was dissected and the major organs (liver, heart) were weighed and the weight of the fetal membranes and number of cotyledons was recorded. Morphologically similar elongated embryos at day 16, chorioallantois with early cotyledon (placentomes) at day 50 and placentomes at day 225 of pregnancy were collected, washed twice in PBS, cut into reasonable pieces if required and stored in RNA later (Ambion Inc, Austin, TX, USA) for later use. Three samples of apparently same morphological quality from each group were used for the present study.


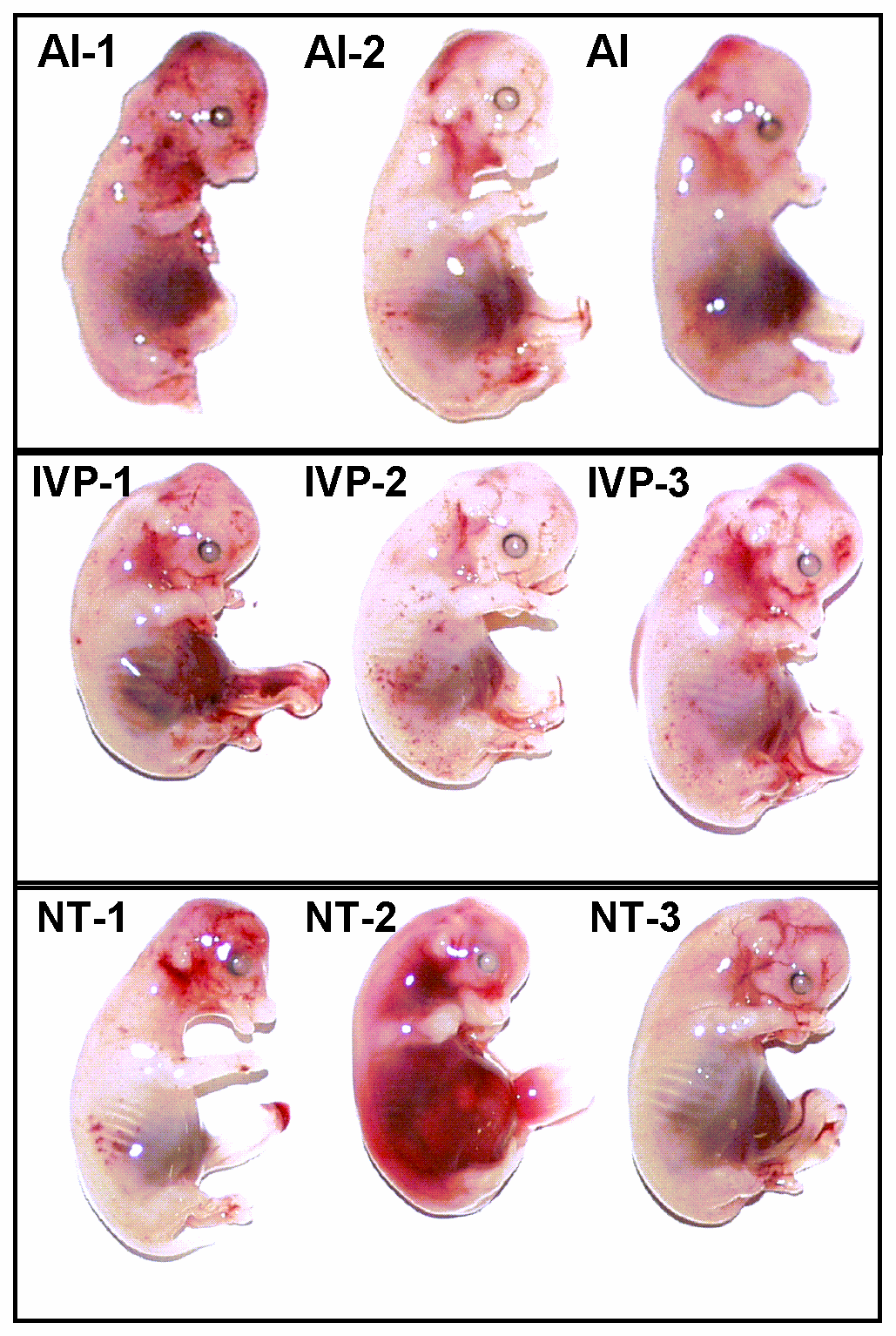


Figure S1. Photograph of fetus at day 50 from the three groups of pregnancy developed by artificial insemination, transferring in vitro produced and somatic cell nuclear transfer embryos.

**Extraction and purification of small RNAs from placenta**

Total RNAs from the three individual frozen placentas (15 mg) of from each group of pregnancy (IVP, NT and AI) and fibroblast cells (4x10^6^) were isolated using miRNeasy mini kit (QIAGEN GmbH, Hilden, Germany). Large (>200 nt) and small RNAs (<200 nt)) were separated using special silica membrane spin column and chemicals of RT^2^ qPCR-Grade miRNA isolation kit (SABioscienecs, Frederick, MD, USA) according to manufacturer’s instructions. For every case, the quality and the concentration of the small RNAs and large RNAs were assessed by NanoDrop 8000 spectrophotometer (NanoDrop, Wilmington, Delaware, USA). Isolated small RNAs were used for the study of expression profiling of 377 individual miRNAs and large RNAs originated from the same sample were used for analysis of regulatory miRNAs processing transcripts in different groups of placenta.

**Genomic DNA, total RNA and protein extraction from placentas and embryos**

To study the global methylation pattern and bisulfite sequencing of promoter of selected gene and quantification of selected transcript at mRNA and protein level, genomic DNA, total RNAs and proteins were isolated from the same sample. Whole individual elongated embryo at day 16, placenta at day 50 (15 mg) and placentomes at day 225 (15 mg) of pregnancy from IVP, NT and AI (at least three of each) were used for isolation using DNA/RNA/Protein purification kit (Norgen Biotek corporation, Thorold, Canada) according to methods recommended by the manufacturer. In addition, 5 early blastocysts, 5 expanded blastocysts (from each IVP, NT and AI method), donor fibroblast cells with serum starvation and without starvation (each in triplicate) were also used to isolate total RNAs using the same procedures. For every cases the quality and the concentration of the nucleic acid was assessed by NanoDrop 8000 spectrophotometer (NanoDrop, Wilmington, Delaware, USA) and subsequent analysis was performed by 2100 Bioanalyzer (AgilentTechnologies, Santa Clara, CA). Protein quantity was assessed by NanoDrop using the absorbance at 280 nm. All these nucleic acids and protein were stored at -80°C in aliquots for the downstream experiments.

**Large scale expression profiling of miRNAs by real-time quantitative PCR**

Total of 166 ng small RNAs from 3 placentas derived from every group of pregnancy at day-50 (IVP, NT and AI) and donor cells (in triplicate) were synthesized into first strand cDNAs using RT^2^ miRNA first strand kit (SABiosciences). Real time qPCR of miRNAs was performed using 384-well miRNAs primed PCR plate (SABiosciences) comprised of 377 individual miRNAs (most of them are conserved in human, mouse and bovine), 4 endogenous controls (U6, Snord44, Snord47 and Snord48), 2 reverse transcription controls and 2 positive PCR controls according to the protocols provided by the manufacturer. The assays were performed in ABI 7900 HT real time PCR system (Applied Biosystems, Foster City, CA, USA) with sybr green technology (SABiosciences). Synthesized cDNAs were diluted 10 times, mixed with 2 ml of 2 X RT^2^ Sybr green PCR master mixes (SABiosciences) and 1.9 ml of ddH_2_O. Mixed cocktail (10 µl per well of 384 well plate) was added and thermal cycling was performed as 95°C for 10 min, 40x of (95°C for 15 sec, 60°C for 40 sec and 72°C for 30 sec). Multichannel laboratory automation workstation was used pipette the mix into 384 well plates precisely (Biomek® NX^P^, Beckman Coulter, Krefeld, Germany). Each individual sample was applied to one 384 well PCR plate and quality of the assay was assessed by the result of control wells and melting curve analysis as recommended. Instrument was set to automatic baseline but threshold value was adjusted manually to 0.045 (above the background signal but within the lower half to one-third of the linear phase of the amplification plot) for all assays performed in the study. Data were analysed by ΔΔC_t_ method and normalization was performed by geometric mean of four endogenous controls through SAbiosciences’s PCR array data analysis on-line web-based analysis portal, which is provided with t test (http://www.sabiosciences.com/pcr/arrayanalysis.php). Expression levels were compared in multiple ways for different group of placenta to find out fold regulation and a fold regulation 2 or more with the value of P less than 0.05 were considered as significant different expression.


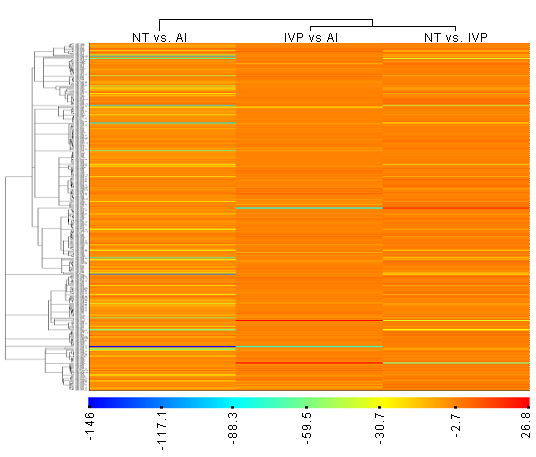


Figure S2. Hierarchical cluster of NT, IVP and AI Day 50 placenta compared to each other and characterization of differentially expressed miRNAs. The log2 fold change value of 377 miRNAs from each comparison is cluster arranged to highlight global differential miRNA expression.

**
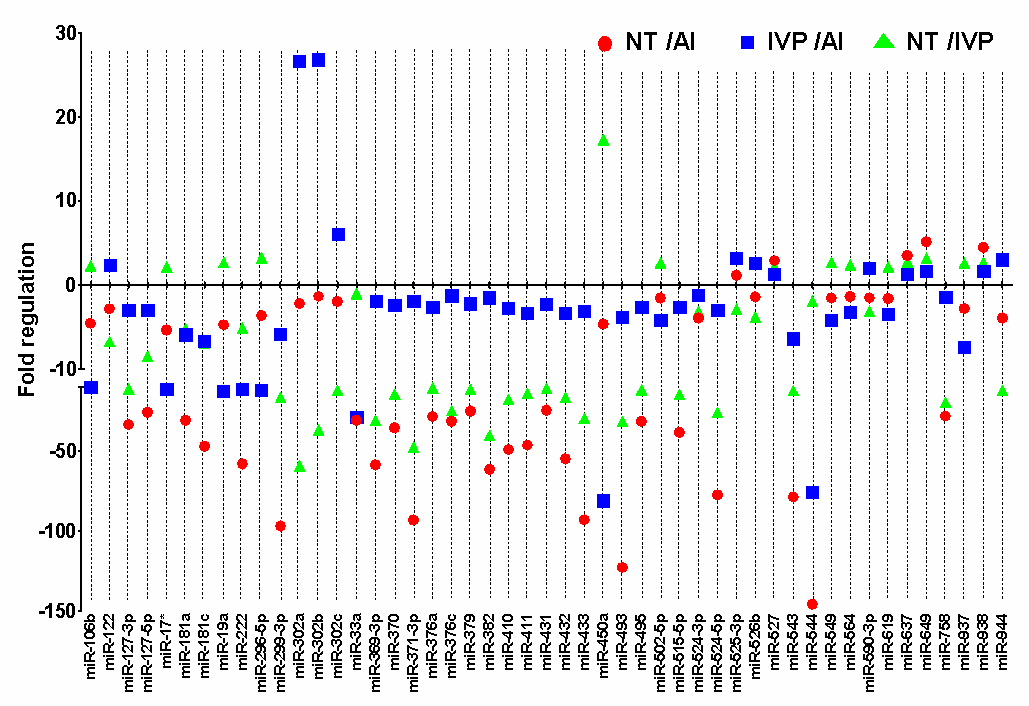
**

Figure S3. Plot diagram of the magnitude of fold regulation of most differentially regulated miRNAs in Day 50 placenta of different sources of pregnancies. The diagram shows the degree of expression difference of 49 miRNAs in Day 50 NT placenta compared to AI (red circle), in Day 50 IVP placenta compared to that of AI (blue rectangle) and in Day 50 NT placenta compared to that of IVP (green triangle).

**Whole mount blastocyst in situ hybridization of miRNAs**

Whole mount in situ hybridization of 10 miRNAs in in vitro, in vivo and NT expanded blastocyst was performed to identify specific expression pattern either in the trophectoderm or inner cell mass. Whole mount in situ hybridization of miRNAs in the expanded blastocysts was performed as described elsewhere [3]. At least 3 embryos were used for the hybridization of each miRNA. According to the expression patterns miRNAs in IVP embryos, selected candidate trophoblast and inner cell mass specific and imprinted miRNAs were localized to the expanded blastocysts derived from AI and nuclear transfer. For hybridization, embryos were rehydrated in series of methanol/PBS, post-fixation (4% paraformaldehyde for 10 minutes), acetylation (2.33 ml triethanolamine, 500 µl acetic anhydride, H_2_O up to 200 ml, readily prepared and treated for 10 minutes) and proteinase K treatment (10 µg/ml, 10 minutes) were carried out, where each step was followed by a 3 times brief wash (10 minutes) in PBS. Two hours of pre-hybridization was performed at 55-59°C in hybridization solution (50% formamide, 5× SSC, 0.1% Tween-20, 50 μg/ml heparin, and 500 mg/ml yeast tRNA). Embryos were incubated overnight with 3'-Digoxigenin (DIG) labeled LNA-modified oligonucleotide probes (1 pM) for mir-31, -96, -127, -215, -222, -223, -299, -320a, -302b,- 431, -450, -544 and let-7d, together with U6 RNAs (Exiqon, Vedbaek, Denmark) in hybridization buffer in a humidified chamber at the temperature 20°C below the Tm of probes. After overnight incubation, embryos were washed briefly in wash buffer (similar to hybridization buffer but without tRNA) and serial wash in 2XSSC/wash buffer (each time 10 minutes) to final three washes in 0.2X SSC each for 30 minutes at hybridization temperature was performed. Blocking, incubation with anti-DIG-AP antibody, washing and color development (Fast Red substrate reaction) was performed as described previously [4]. Embryos were mounted individually with VectaShield containing DAPI (Vector laboratories, Burlingame, CA) and analyzed by confocal laser scanning microscope (CLSM LSM-510, Carl Zeiss, Germany).

**Reverse transcription and SYBR green qPCR for selected miRNAs**

Temporal expression of selected miRNAs has been examined in blastocyst, expanded blastocyst, day-16 elongated embryo, day-50 placenta and day 225 placentome (from AI, IVP and SCNT). All the reagents and kits used for this purpose were obtained from Exiqon (Exiqon, Vedbaek, Denmark). A 36 ng total RNA from each sample was applied to synthesize first strand cDNA using Universal cDNA synthesis kit. Real time qPCR was performed using LNA™ PCR primer set for mir- 21, -24, -127-3p, -135b, -299-5p, -302, -376a, -431, and mir-544a with universal RT primers using SYBR Green master mix in ABI PRISM® 7000 sequence detection system (Applied Biosystems, Foster City, CA, USA). Dilution of cDNA, preparation of mix and thermal cycling condition was performed as recommended by the manufacturer. Data were analyzed as mentioned before except, normalization was performed using the mean Ct value of U6 RNA and 5S ribosomal RNA.

**Reverse transcription and SYBR green qPCR for miRNA processing genes**

Important candidate genes involved in transcription, processing and generating mature miRNAs were quantified in day 50 placenta derived from IVP, AI and NT pregnancy by qPCR. Primers for qRT-PCR analysis (Table 1) were designed using the Primer Express 2.0 software program (Applied Biosystems, Foster City, CA) and synthesized by Eurofins MWG Operon (Ebersberg, Germany). The sequences of PCR primers are listed in Table 1. Reverse transcription of 600 ng total RNA from each sample was performed using Superscript II Reverse Transcriptase (Invitrogen, Carlsbad, CA) in combination with random primers (Invitrogen, Carlsbad, CA) and oligo (DT)_23_(Sigma). The cDNA was stored at -20°C until use. All primers utilized were designed and optimized in order to ensure optimum reaction efficiencies both for target and housekeeping reference genes (GAPDH, Histone). Triplicate reactions were performed for each gene by standard PCR protocol with a 20 µl reaction volume consisting of 10 µl of iTaq SYBR Green Supermix with ROX (Bio-Rad, Hercules, CA), forward and reverse primers at 200-300 nM final concentration and 2 µl diluted template cDNA. A universal thermal cycling parameter specified for the instrument was 50°C for 10 sec, 95°C for 10 min, followed by 40 amplification cycles at 95°C for 15 sec and at 60°C for 1 min. In addition, at the end of the last cycle, dissociation curve was generated by starting the fluorescence acquisition at 60˚C and taking measurements every 7 sec interval until the temperature reached 95˚C. The same PCR protocol was used for all primers and Data was normalized using ΔCt (average Ct for the housekeeping gene minus Ct for the gene of interest) and subsequent analysis was performed using ΔΔCt methods [5]. One-way analysis of variance with tukey test was performed to compare expression of each gene in different types of placenta. A probability of P≤ 0.05 was considered to be significant differentially expressed.

Table S1: **List of miRNA processing genes and primers used in this study**

| Gene |  |  | Sequences (5´-3´) | T_A_ (°C) |
| --- | --- | --- | --- | --- |
| GAPDH | : | Forward | AATGGAAAGGCCATCACCATC | 57 |
|  |  | Reverse | GTGGTTCACGCCCATCACA |  |
| Histone | : | Forward | GCCGTATTCATCGACACCTGA | 55 |
|  |  | Reverse | CTCCACGAATAGCAAGTTGCAA |  |
| ADAR1 | : | Forward | AATGGCTTTGCTGCAGAGTT | 55 |
|  |  | Reverse | GCGCTCTGCTTTCTCTGTTT |  |
| DGCR8 | : | Forward | GGAAGCTGGCAAACAAGATCC | 55 |
|  |  | Reverse | GGTTGGTTTCATGTGCTCGAA |  |
| EIF2C1/AGO1 | : | Forward | AGAGTGGAGTATGCAGTGCTCG | 55 |
|  |  | Reverse | GGGCATCAACATCGTTGTCA |  |
| EIF2C2/AGO2 | : | Forward | AGCGCTGCATTAAGAAGCTGA | 55 |
|  |  | Reverse | CCGTCATGTCATCCTTCACCTT |  |
| EIF2C3/AGO3 | : | Forward | TTCCACACGGGCATTGAGAT | 55 |
|  |  | Reverse | TATTTACAGAAGCATGGCTGGC |  |
| EIF2C4/AGO4 | : | Forward | CAACACCAAGCCACGGAGTAT | 55 |
|  |  | Reverse | GAAATCTTCCGCAGCTGGTCT |  |
| FMR1 | : | Forward | TAGTGGCAGGACAGCGATGTA | 55 |
|  |  | Reverse | TTTAAGGTATGGGTCAGGGCC |  |
| GEMIN4 | : | Forward | TCCCAACAAACCTGCCACA | 55 |
|  |  | Reverse | TCACTGATGGACAAAACCACG |  |
| GEMIN6 | : | Forward | TGGTGGGAAATGATGAAGCAC | 55 |
|  |  | Reverse | GCATGGTTGGACACACATCTG |  |
| GEMIN7 | : | Forward | AGAGCTGAGTGGGTTTGAGCA | 55 |
|  |  | Reverse | TGTTACGCTGACCGCTTTGTA |  |
| POLR2A | : | Forward | ACCTGGACGTGGCCAATTT | 54 |
|  |  | Reverse | AACATATGGAGGCCTGGGAGA |  |
| POLR2G | : | Forward | AACCTGTGCAGGAAACATGA | 54 |
|  |  | Reverse | CTTGGAAGAGTCCACAAGCA |  |
| RNASEN | : | Forward | GATGATTACCTGGGGCTTGT | 55 |
|  |  | Reverse | GGCTGCTAAGCCATAGGAAG |  |
| RANGAP1 | : | Forward | AGGGTCTTCCCATCGATTCT | 55 |
|  |  | Reverse | GCTTGCTCCCTTAAGCAATG |  |
| SIP1 | : | Forward | GGCACAATTTTCGACTGTTCG | 55 |
|  |  | Reverse | AGCCCCTTCAGCACATAACCT |  |
| XPO1 | : | Forward | TCCGACTTGCTCCAACAATGT | 55 |
|  |  | Reverse | CAAGGAACCAATGTGAAGGGA |  |
| XPO4 | : | Forward | CAAGGTACACACGGTCCAAAGA | 55 |
|  |  | Reverse | GGCTCCAAATGTACAAGCCAA |  |
| XPO5 | : | Forward | TCTTTGTGAAGCCTCTGGTG | 55 |
|  |  | Reverse | TGTTCCTCCAGCATCTCTTG |  |

**Global DNA methylation**

Genomic DNA isolated from the 3 elongated day 16 embryos and 3 day 50 placenta from each IVP, AI and SCNT pregnancy was used to quantify the global methylation status using Methylamp Global DNA Methylation Quantification Ultra kit (Epigentek, Brooklyn, NY) according to user instruction provided by the manufacturer. Briefly 200 ng of genomic DNA from each sample was immobilized to the strip well specifically coated with DNA affinity substance. The methylated fraction of DNA was recognized by 5-methylcytosine antibody and quantified through an ELISA-like reaction. Serial dilution of positive control (synthesized polynucleotide methylated at every 5-cytosin) in 6 points (0.4, 1, 2, 5, 10 and 20 ng/well) was used to generate a standard curve. Color was developed and absorbance read was performed in ThermoMax microplate reader (Molecular Devices, Sunnyvale, CA) at 450 nm. Slope was determined as OD/ng by plotting OD value versus amount of positive control. DNA methylation (%) was calculated by using the formula [x 100%] where, 41.7 is the GC content in bovine genomic DNA and Methyl DNA (ng) = {Sample OD- Negative control OD)/slope}.

**Bisulfite genomic sequencing**

Putative promoter region, CpG Island and transcription start site of bovine AGO2 / EIF2C2 in the 5´ untarnslated region (extracted from ENSEMBL genome browser) has been retrieved as commonly predicted region using the criteria [6-8] by multiple freely available software tools namely Promoter 2.0 Prediction Server- http://www.cbs.dtu.dk [9], BDGP Search tools- http://www.fruitfly.org/seq_tools/promoter.html [10], MethPrimer-http://www.urogene.org [11]. The retrieved region has been analyzed for presence of transcription factor binding sites using Genomatix software suite 2.0 (http://www.genomatix.de). The genomic region (ENSEMBL Genome browser 63: Bos Taurus, Btau 4.0, chromosome 14: 2372494-2373020, sense strand) 500 bases upstream to the first exon of AGO2 has been used to design primers for the amplification of bisulfite converted DNA using MethPrimer and Methyl Primer Express® Software v1.0 (Applied biosystem, Foster City, CA). Genomic DNA (500 ng) from the day-50 placenta (N=3) derived from each IVP, AI and SCNT pregnancy has used for bisulfite conversion using EZ DNA Methylation Kit (Zymo Research, Orange, CA) according to manufacturers’ instruction. Each converted DNA was used to amplify the putative promoter region of AGO2 using selected primers (F- GCGAGGAGTTTGTTATTTTAAGAAGTGA, R- CACCTCCAACCCAAAACCAA). Amplified PCR products were purified using QIAquick PCR purification kit (QIAGEN GmbH, Hilden, Germany) and then cloned into the pGEM-T Easy vector (Promega, Mannheim, Germany) and transformed into *E. Coli*. A minimum of 10 different clones from every sample were randomly selected and processed for sequencing with M13 primers using CEQ8000 sequencer system (Beckman Coulter, Brea, CA). Conversion efficiency and methylation sites were analyzed by Quantification Tool for Methylation Analysis [12]. Clones with less than 95% conversion efficiency was discarded from further analysis and the difference between the values was analyzed with t-test (Fisher's exact test) using SAS v.9.2, where *P* ≤ 0.05 was considered to be statistically significant.

**Western blotting**

Total proteins extracted from three Day-50 placentas from each group (IVP, AI and SCNT) were pooled equally (30 µg) and separated by SDS-PAGE (gradient 4-18%) and transferred onto a nitrocellulose membrane (Amersham Biosciences) and blocking was performed in buffer (20 mM Tris pH 7.5, 150 mM NaCl, 0.05% Tween-20 and 1% polyvinylpyrolidone) at room temperature for 1 hour. The membrane was then incubated with goat anti-eIF2C2 polyclonal antibody **(Santa Cruz Biotechnology**, Santa Cruz, CA) in the blocking medium overnight at 4°C. Non-specific binding of antibody was washed off with six changes of 0.1% PBST. The HRP-conjugated mouse anti-goat IgG (Santa Cruz) was used as the secondary antibody. The membrane was incubated for 1 h at room temperature with secondary antibody, followed by washed with six changes of 0.1% PBST. The chemiluminescence was detected by using the ECL plus western blotting detection system (Amersham Biosciences) and visualized by using Kodak BioMax XAR film. GAPDH antibody (Santa Cruz) was used as a loading control. The membrane was stripped by incubation in 2% SDS, 100 mM Tris-HCl and 0.1% beta-mercaptoethanol for 30 min at 60°C and re-probed with GAPDH antibody.

**Supplementary references:**

1. Hölker M, Petersen B, Hassel P, Kues WA, Lemme E, Lucas-Hahn A, Niemann H: **Duration of in vitro maturation of recipient oocytes affects blastocyst development of cloned porcine embryos**. *Cloning Stem Cells* 2005, **7**(1):35-44.

2. El-Sayed A, Hoelker M, Rings F, Salilew D, Jennen D, Tholen E, Sirard MA, Schellander K, Tesfaye D: **Large-scale transcriptional analysis of bovine embryo biopsies in relation to pregnancy success after transfer to recipients**. *Physiol Genomics* 2006, **28**(1):84-96.

3. Hossain MM, Ghanem N, Hoelker M, Rings F, Phatsara C, Tholen E, Schellander K, Tesfaye D: **Identification and characterization of miRNAs expressed in the bovine ovary**. *BMC Genomics* 2009, **10**:443.

4. Obernosterer G, Martinez J, Alenius M: **Locked nucleic acid-based in situ detection of microRNAs in mouse tissue sections**. *Nat Protoc* 2007, **2**(6):1508-1514.

5. Livak KJ, Schmittgen TD: **Analysis of relative gene expression data using real-time quantitative PCR and the 2(-Delta Delta C(T)) Method**. *Methods* 2001, **25**(4):402-408.

6. Fatemi M, Pao MM, Jeong S, Gal-Yam EN, Egger G, Weisenberger DJ, Jones PA: **Footprinting of mammalian promoters: use of a CpG DNA methyltransferase revealing nucleosome positions at a single molecule level**. *Nucleic Acids Res* 2005, **33**(20):e176.

7. Gardiner-Garden M, Frommer M: **CpG islands in vertebrate genomes**. *J Mol Biol* 1987, **196**(2):261-282.

8. Saxonov S, Berg P, Brutlag DL: **A genome-wide analysis of CpG dinucleotides in the human genome distinguishes two distinct classes of promoters**. *Proc Natl Acad Sci U S A* 2006, **103**(5):1412-1417.

9. Knudsen S: **Promoter2.0: for the recognition of PolII promoter sequences**. *Bioinformatics* 1999, **15**(5):356-361.

10. Reese MG: **Application of a time-delay neural network to promoter annotation in the Drosophila melanogaster genome**. *Comput Chem* 2001, **26**(1):51-56.

11. Li LC, Dahiya R: **MethPrimer: designing primers for methylation PCRs**. *Bioinformatics* 2002, **18**(11):1427-1431.

12. Kumaki Y, Oda M, Okano M: **QUMA: quantification tool for methylation analysis**. *Nucleic Acids Res* 2008, **36**(Web Server issue):W170-175.
